# Nitrogen Limitation and Slow Drying Induce Desiccation Tolerance in Conjugating Green Algae (Zygnematophyceae, Streptophyta) from Polar Habitats

**DOI:** 10.1371/journal.pone.0113137

**Published:** 2014-11-14

**Authors:** Martina Pichrtová, Jana Kulichová, Andreas Holzinger

**Affiliations:** 1 Charles University in Prague, Faculty of Science, Department of Botany, Prague, Czech Republic; 2 Academy of Sciences of the Czech Republic, Institute of Botany, Třeboň, Czech Republic; 3 University of Innsbruck, Institute of Botany, Functional Plant Biology, Innsbruck, Austria; Institute of Genetics and Developmental Biology, Chinese Academy of Sciences, China

## Abstract

**Background:**

Filamentous Zygnematophyceae are typical components of algal mats in the polar hydro-terrestrial environment. Under field conditions, they form senescent vegetative cells, designated as pre-akinetes, which are tolerant to desiccation and osmotic stress.

**Key Findings:**

Pre-akinete formation and desiccation tolerance was investigated experimentally under monitored laboratory conditions in four strains of Arctic and Antarctic isolates with vegetative *Zygnema* sp. morphology. Phylogenetic analyses of *rbc*L sequences revealed one Arctic strain as genus *Zygnemopsis*, phylogenetically distant from the closely related *Zygnema* strains. Algae were cultivated in liquid or on solidified medium (9 weeks), supplemented with or lacking nitrogen. Nitrogen-free cultures (liquid as well as solidified) consisted of well-developed pre-akinetes after this period. Desiccation experiments were performed at three different drying rates (rapid: 10% relative humidity, slow: 86% rh and very slow); viability, effective quantum yield of PS II, visual and ultrastructural changes were monitored. Recovery and viability of pre-akinetes were clearly dependent on the drying rate: slower desiccation led to higher levels of survival. Pre-akinetes survived rapid drying after acclimation by very slow desiccation.

**Conclusions:**

The formation of pre-akinetes in polar *Zygnema* spp. and *Zygnemopsis* sp. is induced by nitrogen limitation. Pre-akinetes, modified vegetative cells, rather than specialized stages of the life cycle, can be hardened by mild desiccation stress to survive rapid drying. Naturally hardened pre-akinetes play a key role in stress tolerance and dispersal under the extreme conditions of polar regions, where sexual reproduction and production of dormant stages is largely suppressed.

## Introduction

Green microalgae, despite being widely considered aquatic organisms, occur often in hydro-terrestrial or aero-terrestrial environments [1], [2], and their exceptional ability to adapt to life on land is well-known. The transition to land has appeared independently many times within this group and aeroterrestrial algae are found in various green algal lineages [3], [4]. The most important land colonization event in the Earth's history appeared within streptophytes, in the ancestor of all land plants (Embryophyta), which occurred during the Ordovician Period [5]. There is an ongoing discussion concerning which of the algal streptophyte classes is the actual sister group to Embryophytes; currently, either Zygnematophyceae or a clade consisting of Zygnematophyceae and Coleochaetophyceae are considered the closest algal relatives of land plants [6]–[9].

Zygnematophyceae, the most species-rich lineage of streptophyte algae, are filamentous or unicellular microalgae that do not form any flagellate stages and reproduce sexually by conjugation, producing a dormant zygospore [10], [11]. Many Zygnematophyceae are found in conditions that expose them to dehydration stress, such as those found in aeroterrestrial habitats [12], airborne particles [13], and surface of glaciers [14], [15]. Filamentous Zygnematophyceae are also typically found in ephemeral pools or streams that are subject to occasional or regular desiccation. Such algal mats are a noticeable feature of polar tundra habitats in particular, where they are among the most important primary producers. The genus *Zygnema* has been repeatedly reported from the Arctic and Antarctic [16]–[21], but conjugation and zygospore formation have been reported there only once – in *Zygnema* cf. *leiospermum* from Central Ellesmere Island in Canadian Arctic [22].

Mechanisms of desiccation tolerance in green algae have been reviewed recently [2], [23], [24]. Certain algae are considered desiccation-tolerant, but their actual survival capability is dependent upon the conditions under which desiccation occurs. Various factors significantly influence recovery after dehydration: for example, the duration of desiccation [25], light availability during desiccation [26], and relative air humidity, which corresponds with the drying rate [25], [27].

Green microalgae can rarely fully withstand desiccation in the vegetative state; this ability has been found in some desert species, mostly in those belonging to the Chlorophyte lineage [26]. More often they form specialized cells, including zygospores, aplanospores or akinetes, which are capable of surviving periods of desiccation. Akinetes are old (senescent), stationary phase cells rather than morphologically specialized dormant stages. However, they have been found to be much more stress-resistant than young, actively growing vegetative cells. They usually possess thick cell walls and are filled with storage materials [28]–[30]. The formation of such stress-resistant stationary phase cells by *Zygnema* sp. has been observed under both experimental [28], [31]–[33] and field conditions [18], [21], [34]. Here, we term such old cells “pre-akinetes”, in accordance with the published literature [16], [21], [32]. The term “akinete” refers in *Zygnema* spp. to specialized cells with distinctive cell-wall characteristics, similar to that of the zygospores. This terminology and the differences between both types of cells have been recently addressed [21], [32].

The formation of pre-akinetes in old cultures and their role in desiccation tolerance was demonstrated in a study of *Zygnema* sp. from Texas [28] and recently in a parallel study of two strains of *Zygnema* sp. isolated from alpine regions [33]. The ageing of cultures is most likely the result of nitrogen-starvation treatment, as nutrients are depleted by intensive growth under batch culture conditions. During nitrogen starvation, protein synthesis is suppressed and metabolism shifts to the production of carbohydrates and lipids, which are substances of great biotechnological interest [30], [35], [36]. The occurrence of storage product-filled cells in Arctic and Antarctic *Zygnema* sp. has been frequently reported in field samples [16], [18], [21]. Recently, the formation of pre-akinetes was observed under natural conditions in the Arctic and their resistance to osmotic stress was found to be significantly related to their natural hydration status [21]. The authors concluded that the experience of mild dehydration stress was crucial for pre-akinete hardening, and hypothesized that other environmental factors (possibly nutrient starvation) must be involved in their initial formation [21].

In this study, we test this hypothesis; four strains, isolated from putative *Zygnema* sp. mats growing in polar regions that readily produced pre-akinete stages, were used for the experiments. First, we studied the effects of mild dehydration stress and nitrogen starvation on the formation of pre-akinetes. Next, desiccation stress resistance of pre-akinetes was investigated under various rates of drying. Photosynthetic activity during the experiments was assessed regularly by measuring chlorophyll fluorescence. As no conjugating stages were observed, which makes it impossible to determine species according to their morphological traits, the strains were also characterized by their *rbc*L sequences. Finally, we used electron microscopy to characterize the ultrastructure of pre-akinetes before and after desiccation.

## Materials and Methods

### Cultivation of algal material and light microscopy observations

Four strains (B, C, E and L) with a vegetative *Zygnema* morphology were used for the experiments. Capital letters were assigned to the strains in accordance with previously published studies on polar *Zygnema* spp. [31], [37]. *Zygnema* sp. B (CCALA 976) was isolated in 2010 from a shallow seepage pool on Svalbard (Arctic, [37]). Strain L was isolated in 2011 from the same locality in Svalbard (Arctic) as *Zygnema* sp. B and was deposited in the same culture collection, the Culture Collection of Autotrophic Organisms in Třeboň, Czech Republic (CCALA, www.butbn.cas.cz/ccala/index.php; accession number CCALA 1068). No specific permits were required for the field studies in Svalbard. The Svalbard Act of 15 June2001 No. 79 Relating to the Protection of the Environment in Svalbard, states: ‘The collection of flora for research or teaching purposes is permitted where this does not make significant inroads into the local populations of the flora involved.’ We confirm that the field studies did not involve endangered or protected species. *Zygnema* sp. C was also obtained from CCALA, (strain No. 880). This strain has been previously isolated by J. Elster and J. Šnokhousová in 2008 from a sample originally collected from a seepage reaching Monolith Lake on James Ross Island, Antarctica. *Zygnema* sp. E was obtained from the culture collection of Cryophilic Algae, Fraunhofer IZI, Potsdam-Golm, Germany (CCCryo 278-06), it was previously isolated in 2006 from a meltwater pool north of Artigas Base freshwater lake (also known as Lago Uruguay or Lake Profound), Fildes Peninsula, Maxwell Bay, King George Island, South Shetland Islands, Antarctica.

**Figure 1:**
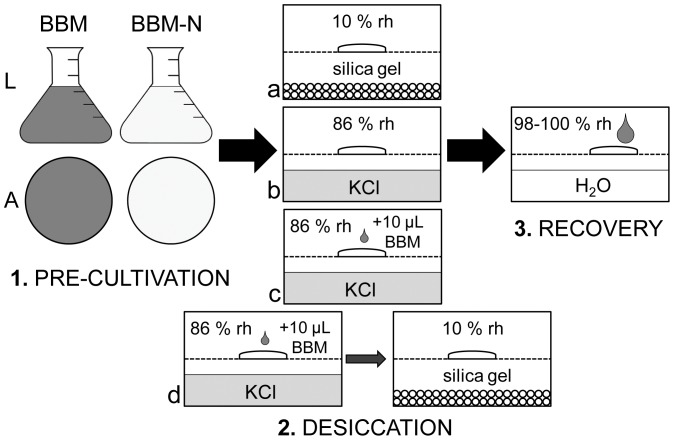
Schematic illustration of the experimental design. Four combinations of pre-cultivation conditions, four desiccation regimes (a–d; d applied only on samples pre-cultivated on agar) and final recovery after rewetting. For details see methodology section of the text.

Cultures of each of the four strains were transferred in the exponential growth phase into either standard Bold's Basal medium (BBM) [38] or into BBM lacking nitrate (termed henceforth ‘BBM’ and ‘BBM-N’, respectively). The cultures were grown in 6-well microplates in either liquid (‘L’) medium or on medium solidified with 1.5% agar (‘A’). Thus, four combinations of pre-cultivation conditions for each strain were achieved (Fig. 1); these are henceforth referred to as ‘A BBM’, ‘A BBM-N’, ‘L BBM’ and ‘L BBM-N’. The plates were kept under optimal growth conditions of 20°C and continuous light (intensity: 35 µmol m^−2^ s^−1^) for 9 weeks.

Light microscopy of the algal strains was undertaken using a Zeiss Axiovert 200 M light microscope (Carl Zeiss AG, Oberkochen, Germany) equipped with a Zeiss Axiocam MRc5 camera. The cultures were investigated after 9 weeks of pre-cultivation. Further observations using light microscopy were made following the desiccation experiment and 48 hours of subsequent rehydration, when the proportion of surviving cells was estimated.

### DNA isolation and phylogenetic analysis

DNA was isolated using the Invisorb Spin Plant Mini kit (Invitek, Germany), according to the manufacturer's instructions. The chloroplast-encoded gene *rbc*L was amplified using the polymerase chain reaction (PCR) and primers RH1 and 1385R [39]. Each reaction contained 13.9 µl sterile Milli-Q water, 2 µl MgCl_2_ (25 mM), 2 µl AmpliTaq Gold 360 Buffer (Applied Biosystems, Carlsbad, CA, USA), 0.4 µl dNTP mix (10 mM), 0.25 µl each primer (25 pmol/µl), 0.2 µl AmpliTaq Gold 360 DNA Polymerase, and 1 µl DNA (10 ng/µl). The PCR programme consisted of an initial denaturation for 10 minutes at 95°C, followed by 35 amplification cycles, each with a 1 minute denaturation step at 94°C, 1 minute annealing at 48°C. and 2.5 minutes extension at 72°C, and a final extension stage at 72°C for 10 minutes. Finally, the PCR products were purified using the GenElute PCR Clean-Up kit (Sigma-Aldrich, USA), according to the manufacturer's protocol, and sequenced by Macrogen Inc. (Seoul, South Korea). The GenBank accession numbers for the sequences are xxx (*Zygnema* sp. B), xxx (*Zygnema* sp. C), xxx (*Zygnema* sp. E) and xxx (*Zygnemopsis* sp. L).

Published sequences from GenBank database were selected on the basis of recent *Zygnema*/Zygnematophyceae phylogenies [40], [41]. The resulting alignment comprised 26 sequences, each of which was up to 1292 nucleotides long.

Three different phylogenetic analyses were performed: maximum likelihood (ML), weighted parsimony (wMP, character weighting), and Bayesian inference (BI). For ML and BI, the sequence evolution model was determined as GTR+I+gamma by MrModelTest 2.3 [42] using the Akaike Information Criterion. The BI phylogenetic trees were constructed using MrBayes 3.2.1 [43]. Two parallel Markov chain Monte Carlo runs were carried out for 3×10^6^ generations, each with one cold and three heated chains. Convergence of the two cold chains was checked by the average standard deviation of split frequencies: the value was 0.001865. Trees and parameters were sampled every 100 generations, and trees from the initial 100 generations were discarded using the sumt burnin function. The BI tree was rooted using the midpoint root function in FigTree 1.3.1 [44]. Bootstrap analyses (ML, wMP) were calculated using Garli 2.0 [45] and PAUP* Portable version 4.0b10 [46]. ML analyses consisted of rapid heuristic searches (100 pseudo-replicates) using automatic termination (genthreshfortopoterm command set to 100,000). The wMP bootstrapping was performed using heuristic searches with 100 random sequence addition replicates, tree bisection reconnection swapping, and random addition of sequences (the number was limited to 10,000 for each replicate).

### Desiccation stress treatments

Initially, desiccation stress resistance was compared between well-developed pre-akinetes from cultures grown on A BBM-N and morphologically diverse cultures grown on A BBM. The experiments followed a standardized setup developed for monitoring physiological performance during controlled dehydration and rehydration [47]. Small samples of each of the four different strains were placed on glass fibre filters (Whatman GF/C). Four of these filters were placed on a perforated metal grid inside a 200 ml polystyrene box sealed with a transparent lid prior to experimental drying. Different drying rates were achieved by desiccating samples inside the containers at different relative humidities (rh): rate 1, a rapid rate of drying, at approximately 10% rh was achieved using 100 g of freshly dried silica gel (Silica Gel Orange, Carl Roth, Karlsruhe, Germany), whereas rate 2, a slow drying rate at approximately 86% rh, was achieved using saturated KCl solution. For generating a very slow rate of drying (rate 3), each biomass sample was wetted with 10 µl L BBM medium prior to desiccation at 86% rh. Additionally, another set of samples, desiccated in this ‘very slow’ mode for 12 hours, was put over silica gel for a further 24 hours. A synoptic scheme of the experimental design is presented in Fig. 1.

After desiccation for 24 hours, the samples were rehydrated by the addition of 0.5 ml fresh L BBM medium and placed into new polystyrene boxes with 100 ml tap water to ensure an environment with very high relative humidity (98–100%). The filters were sprayed regularly with sterile distilled water to prevent additional desiccation stress and allowed to recover for 72 hours. During the experiments, the containers were kept at ambient room temperature under continuous illumination (light intensity: 50 µmol m^−2^ s^−1^).

Samples from liquid cultures (L BBM and L BBM-N) were also desiccated under the three drying rates described above to determine whether pre-akinetes developing under such conditions (i.e., without exposure to mild desiccation stress on agar) reacted to experimental desiccation in a similar manner to those grown on agar.

### Measurement of the effective quantum yield

The effective quantum yield of photochemical energy conversion in PSII (*Φ*_PSII_) [48] was measured using a PAM 2500 fluorometer (Heinz Walz GmbH, Effeltrich, Germany). *Φ*_PSII_ is a relative parameter computed as (F_M_′-F)/F_M_′, where F is steady state fluorescence in the light-adapted state and F_M_′ the maximum fluorescence in the light-adapted state measured after the application of a saturation pulse. Measurements of *Φ*_PSII_ were performed directly through the closed transparent exposure chambers with a constant distance of 5 mm between the probe and the samples. The first measurement was taken immediately after placing the filters into the desiccation chambers; subsequent measurements were then made every 2 minutes for samples desiccated at 10% rh, every 5 minutes for samples desiccated at 86% rh or every 60 minutes for samples undergoing very slow desiccation. Measurement continued until the values of *Φ*_PSII_ reached zero or settled at an above-zero value. The effective quantum yield was also measured 1, 6, 12, 24, 48 and 72 hours following rehydration.

### Transmission electron microscopy

For transmission electron microscopy (TEM), samples of all cultures were taken from cultures pre-grown on A BBM-N, either immediately or following desiccation at 86% rh for 2.5 hours. Samples were fixed according to the methodology described previously [18] with the following modifications. Briefly, samples were fixed for 1 hour in 2.5% glutaraldehyde, 20 mM caccodylate buffer (pH = 6.8), rinsed, and then post-fixed in the same buffer containing 1% OsO_4_ at 4°C for 18 hours. Samples were then dehydrated in increasing concentrations of ethanol and transferred *via* propylene oxide to modified Spurr's resin [49]. Ultrathin sections (∼60 nm) were prepared with a Reichert ultracut microtome, counterstained with uranyl acetate and Reynold's lead citrate, and viewed using a Zeiss Libra 120 transmission electron microscope. Digital images were captured with a ProScan 2k SSCCD camera controlled by OSIS iTEM software, and further processed using Adobe Photoshop (7.0) software.

### Statistical analyses

All measurements of the effective quantum yield were made using four independent replicate samples per strain. The differences in *Φ*_PSII_ (the initial values before desiccation) between individual strains and pre-cultivation conditions were tested separately for agar and liquid cultures by general linear model (GLM) two-way factorial analysis of variance (ANOVA). The factors tested (‘strain’ and ‘nitrogen’) were regarded as fixed effects. Factor levels were compared using Tukey's post-hoc tests. The ANOVA was performed in STATISTICA 10 for Windows.

SIGMAPLOT 9.01 and Adobe Photoshop (7.0) were used for the graphical elaboration of the results showing performance of the cultures during desiccation and subsequent recovery.

## Results

### Phylogenetic analyses

**Figure 2:**
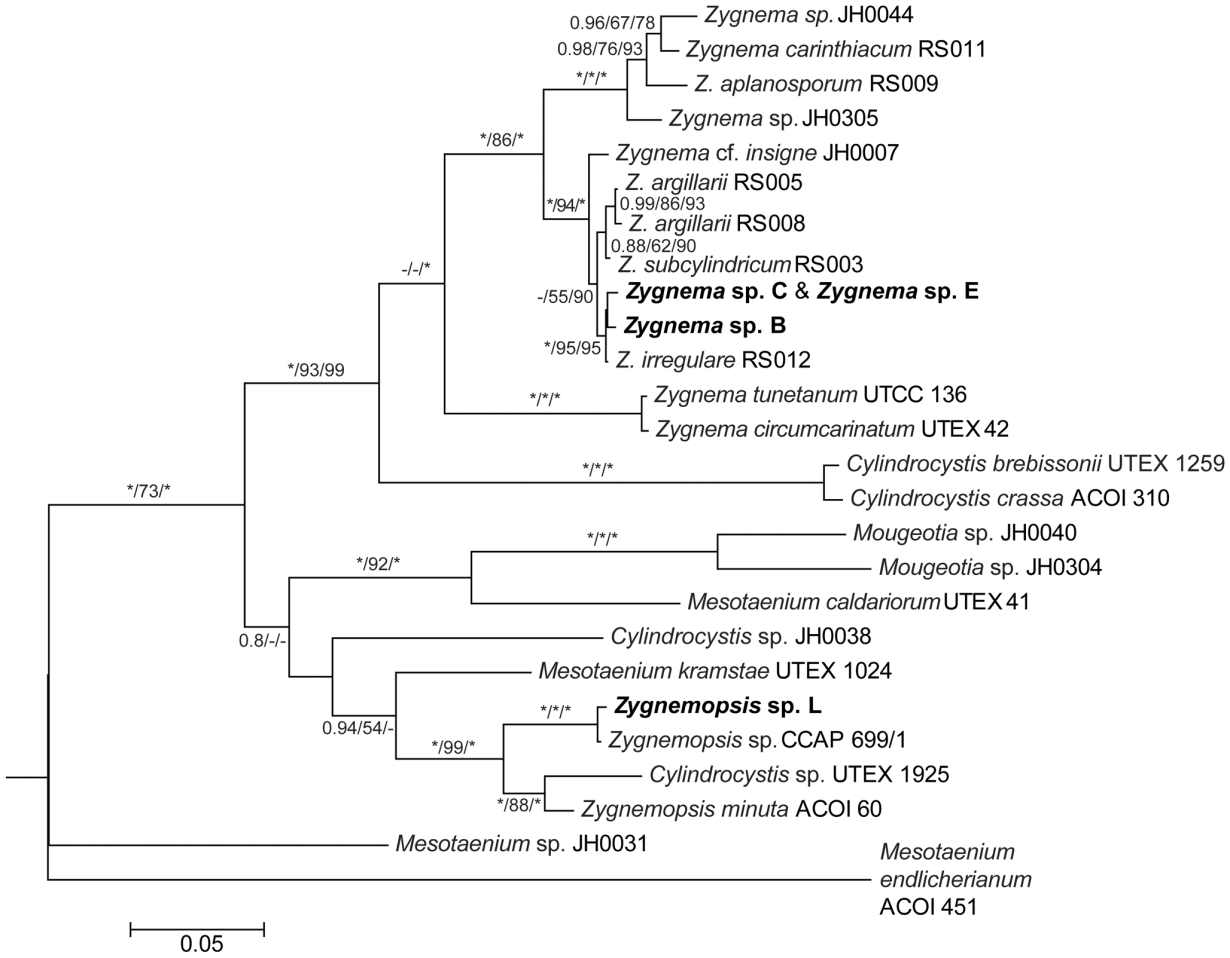
Phylogenetic tree of Zygnematophyceae showing the positions of the strains investigated in this study. A midpoint-rooted Bayesian tree of *rbc*L sequences is shown. Values at the branches indicate Bayesian posterior probabilities (BI PP), maximum likelihood (ML), and maximum parsimony (MP) bootstrap values (BS). Asterisks indicate BI PP = 1.00, and ML and MP BS = 100; dashes indicate BI PP<0.8, and ML and MP BS<50. Strains used in this study are in bold.

Phylogenetic analyses (Fig. 2) of *rbc*L sequences revealed that strains B, C and E were members of the genus *Zygnema* but strain L belonged to another genus, *Zygnemopsis*.

The Antarctic strains, C and E, shared an identical *rbc*L sequence and were closely related to *Zygnema* sp. B from the Arctic. All these three *Zygnema* strains formed a well-supported cluster (BI/ML/MP: 1.0/95/95) together with the strain *Zygnema irregulare* (RS012) [41]. The *rbc*L sequences within this group differed from each other by, at most, seven base-pair substitutions. Species names could not be assigned to any of our strains as no zygospores were observed. Moreover, exact species determination was not possible even with the knowledge of *rbc*L sequences, because sequences of only few species are available and the traditional morphological species concept has not been revised using molecular data yet.

The strain *Zygnemopsis* sp. L fell into a separate *Zygnemopsis* clade (1.0/99/100) and was most closely related to the strain *Zygnemopsis* sp. CCAP 699/1, isolated by Ott in 1965 from an unspecified freshwater habitat in the USA. Sequences of these two strains differed at four sites.

### Investigations using light microscopy

**Figure 3:**
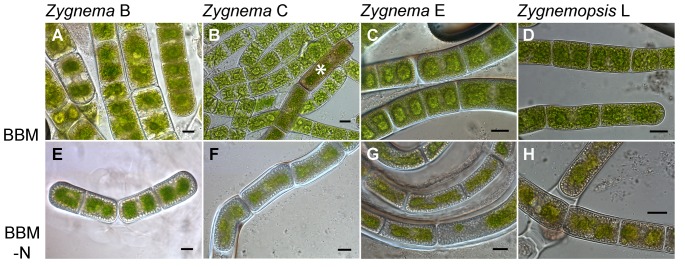
Light micrographs of the strains pre-cultivated on agar medium for 9 weeks. **A–D:** cultures grown on regular BBM medium (A BBM); **E–H:** cultures grown on BBM without nitrate (A BBM-N). The images were taken prior to the desiccation experiments; a *Zygnema* akinete with a distinct brown mesospore is marked with an asterisk. Scale bars: 10 µm.

Samples of algal cultures were observed after 9 weeks pre-cultivation in four combinations of culture conditions. The cultures grown on A BBM showed high morphological variation. The whole range of cell types, from normal vegetative cells to stationary phase cells (pre-akinetes), appeared in all *Zygnema* strains (Fig. 3A–C). It was difficult to perform an exact quantification of different cell types because of the gradual transitions in morphology. Nevertheless, in all cultures of *Zygnema* sp. (B, C and E) more than 50% of cells showed a pre-akinete morphology. In cultures of strains C and E, pre-akinetes with very thick cell walls appeared, but these made up for less than 1% of cells. In *Zygnemopsis* sp. strain L, around 30% of the cells were dead following the pre-cultivation period and the remainder were morphologically homogenous (Fig. 3 D). Very rarely, we also observed akinetes with distinctly colored cell walls, but these appeared only in strain C after A BBM pre-treatment (Fig. 3B).

By contrast, the cells from all the starved (A BBM-N) cultures had a more homogenous appearance: all viable cells were well-developed pre-akinetes filled with storage material that appeared as large globular hyaline inclusions (Fig. 3E–H). The chloroplasts of these cells were small, lacked the typical stellar shape, and were rather yellowish in color, when compared with cells from A BBM cultures. Cells with very thick cell walls appeared occasionally in starved cultures; this was observed in strains C and E, in particular (Fig. 3F and G). Moreover, algal filaments were enveloped by mucilaginous sheaths, which was also apparent macroscopically in cultures grown on agar plates.

A similar effect of nitrogen starvation on cell morphology was observed in cultures grown in liquid media (L BBM, L BBM-N; Fig. S1). Nitrogen-starved cultures consisted only of pre-akinetes whereas cultures grown in L BBM medium also contained vegetative cells with bright-green colored chloroplasts and no distinct storage inclusions.

### Physiological performance during desiccation

**Figure 4:**
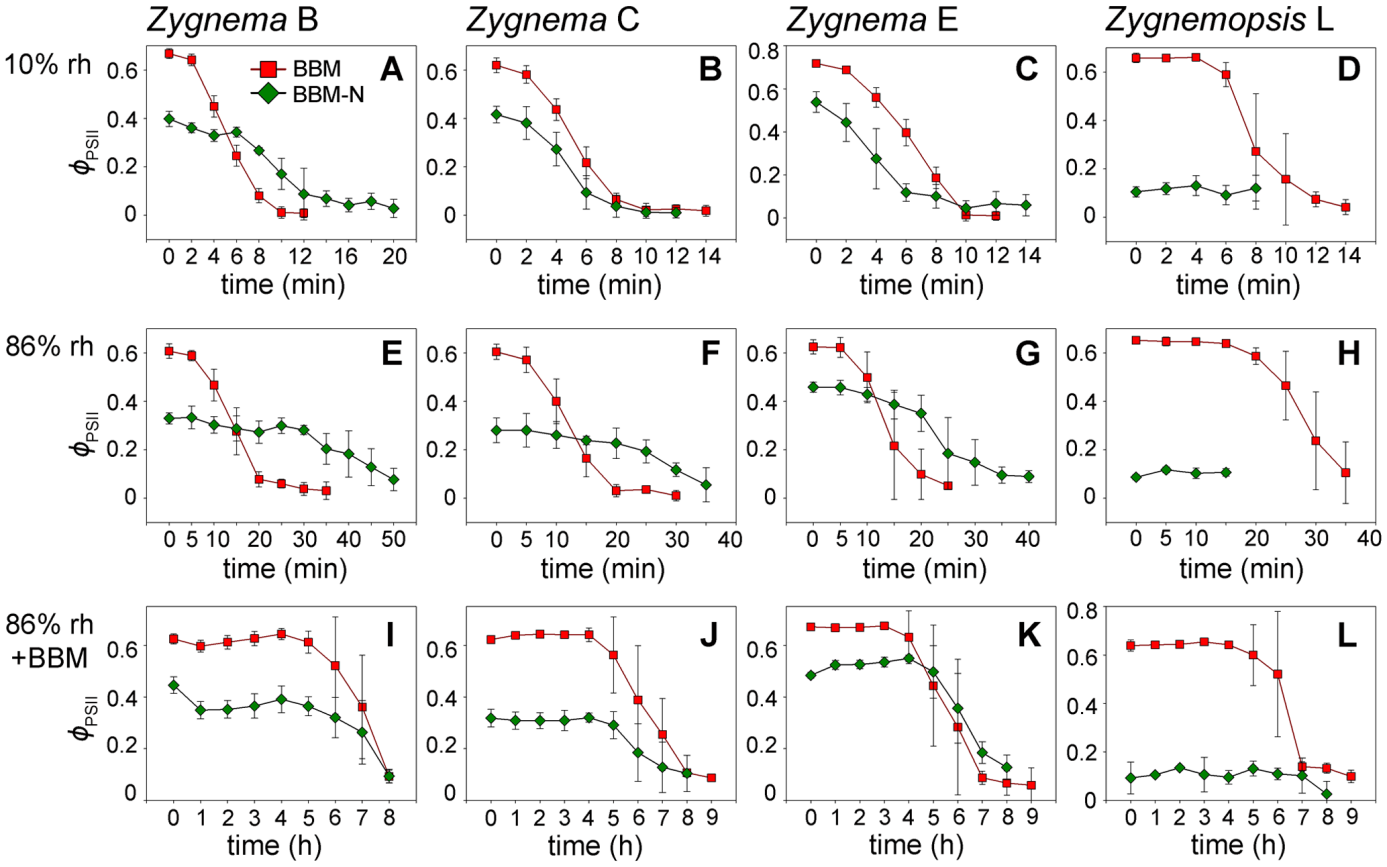
Effective quantum yield (*Φ*_PSII_) under different desiccation scenarios. **A**–**D:** rapid desiccation, approximately 10% relative humidity (rh); **E**–**H:** slow desiccation, 86% rh; **I**–**L:** very slow desiccation, 86% rh plus additional moistening of samples with 10 µl BBM. All strains shown were pre-cultivated on regular agar medium (A BBM) or on medium without nitrate (A BBM-N). Results are means ± standard deviations of four independent experimental replicates.

The initial values of *Φ*_PSII_ differed between the A BBM and A BBM-N cultures for each strain and were always lower in the starved culture (GLM ANOVA, Tukey's post-hoc tests, n = 12, p<0.001; Fig. 4). Following the transfer into the desiccation chambers, *Φ*_PSII_ started to decrease and the rate of the decline differed according to the desiccation scenario. Over silica gel (rh around 10%), the samples began to desiccate almost immediately and *Φ*_PSII_ dropped to zero within 10–20 minutes, indicating the complete cessation of physiological activity (Fig. 4A–D). By contrast, it took up to 50 minutes for the *Φ*_PSII_ to reach its lowest value in samples desiccated at 86% rh (Fig. 4E–H). When the samples were moistened with 10 µl L BBM medium prior to desiccation at 86% rh, the *Φ*_PSII_ remained unchanged for several hours before the values began to drop, with the lowest values being reached as late as 8–9 hours after the beginning of the experiment (Fig. 4I–L). Markedly, in some strains, cultures pre-grown on A BBM lost their physiological activity much faster than those pre-cultivated on A BBM-N (Fig. 4A, E, F, G and K). *Zygnemopsis* sp. L cultivated on A BBM-N showed very small *Φ*_PSII_ values (around 0.1), even prior to any experimental manipulation, indicating the very low performance of this strain under starvation conditions. These initial values did not change, regardless of the desiccation regimes applied (Fig. 4D, H and L).

Further experiments with liquid cultures (L BBM and L BBM-N) revealed very similar responses. The initial values of *Φ*_PSII_ were also significantly lower in nitrogen-starved samples for each strain (GLM ANOVA, Tukey's post-hoc tests, n = 12, p<0.001) and the rate of decline in physiological activity again correlated with the drying rate.

### Physiological performance and viability after rehydration

**Figure 5:**
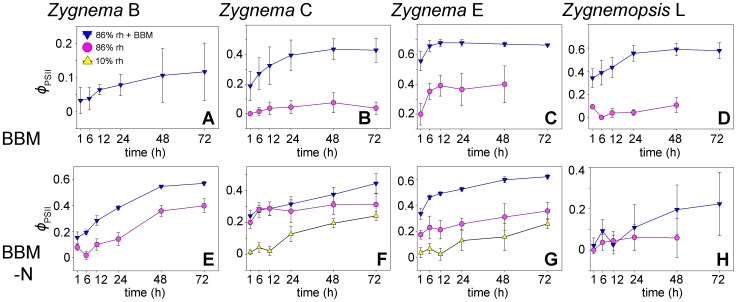
Recovery of effective quantum yield (*Φ*_PSII_) during rehydration after 24 hours desiccation. Each panel compares the performance of the same culture desiccated under different conditions; no plots were constructed for those regimes that were lethal for all of the cells. Samples were collected from cultures pre-cultivated on agar media: **A**–**D:** cultures grown on regular agar medium (A BBM); **E**–**H:** cultures grown on BBM without nitrate (A BBM-N). Results are means ± standard deviations of four independent experimental replicates.

The values of *Φ*_PSII_ started to recover following rewetting of cultures. Noticeably, the recovery rate differed with regard to individual strains, desiccation scenarios, and pre-treatments (Fig. 5, Table 1). In accordance with the recovery rate, the proportion of surviving cells (observed 48 hours after rehydration) also varied (Table 2): strains C and E, pre-cultivated on A BBM-N, survived even very quick desiccation at 10% rh; however, only a small proportion of cells were viable 48 hours after rehydration (Table 2). The samples that had been desiccated at 86% rh showed much better survival capacity: only strain B, pre-treated on A BBM, entirely failed to survive this treatment. All cultures, however, survived the very slow desiccation regime (desiccation at 86% rh plus additional moistening with 10 µl L BBM). The recovery of photosynthetic activity was also fastest after this treatment (Fig. 5, Table 1).

**Table 1:** Relative values of the effective quantum yield during recovery of algal samples pre-cultivated on agar (A BBM and A BBM-N cultures).

Strain		B		C		E		L	
Culture type		A BBM	BBM-N	BBM	BBM-N	BBM	BBM-N	BBM	BBM-N
t = 0	10% rh	ND	ND	ND	2%	ND	7%	ND	ND
	86% rh	ND	25%	0%	70%	32%	39%	14%	0%
	86% rh+BBM	5%	35%	30%	76%	83%	70%	53%	18%
t = 48 h	10% rh	ND	ND	ND	47%	ND	30%	ND	ND
	86% rh	ND	110%	12%	110%	64%	68%	16%	79%
	86% rh+BBM	17%	123%	70%	118%	100%	124%	92%	74%

The given percentages were computed as ratios between initial values of *Φ*_PSII_ and the values measured immediately after rehydration (t = 0) or after 48 hours of rehydration (t = 48 h). No data are shown for those regimes that were lethal for all of the cells (ND). Values shown are means of four, independent experimental replicates.

**Table 2:** Viability of cultures pre-cultivated on agar following 48 hours of rehydration in water (A BBM and A BBM-N cultures).

Strain	B		C		E		L	
Culture type	BBM	BBM-N	BBM	BBM-N	BBM	BBM-N	BBM	BBM-N
10% rh	−	−	−	++	−	+	−	−
86% rh	−	++	+	+++	+++	+++	+	+
86% rh+BBM	+	+++	++	+++	+++	+++	+++	+
86% rh+BBM = >10% rh	+	+	++	+++	+++	+++	−	−

−: No living cells observed; +: <5% living cells; ++: 5–50% living cells; +++: 50–100% living cells.

When samples were exposed first to the very slow desiccation regime for 12 hours and then transferred to 10% rh for additional 24 hours, all *Zygnema* strains produced at least a small proportion of surviving cells (Table 2); however, no cells of *Zygnemopsis* sp. L survived following exposure to 10% rh, even under these modified conditions (Table 2).

An additional set of desiccation experiments using liquid cultures gave very similar results (Table S1). Similar to the samples pre-cultivated on agar, all the samples grown in liquid medium survived the very slow desiccation regime. In addition, all the liquid cultures, except *Zygnema* sp. B (L BBM) and *Zygnemopsis* sp. L (L BBM-N), survived desiccation at 86% rh. However, in contrast to the agar cultures, no liquid culture survived desiccation at 10% rh, indicating that pre-cultivation on a solidified medium improves the acquisition of desiccation tolerance.

### Ultrastructural investigation of starved cultures

**Figure 6:**
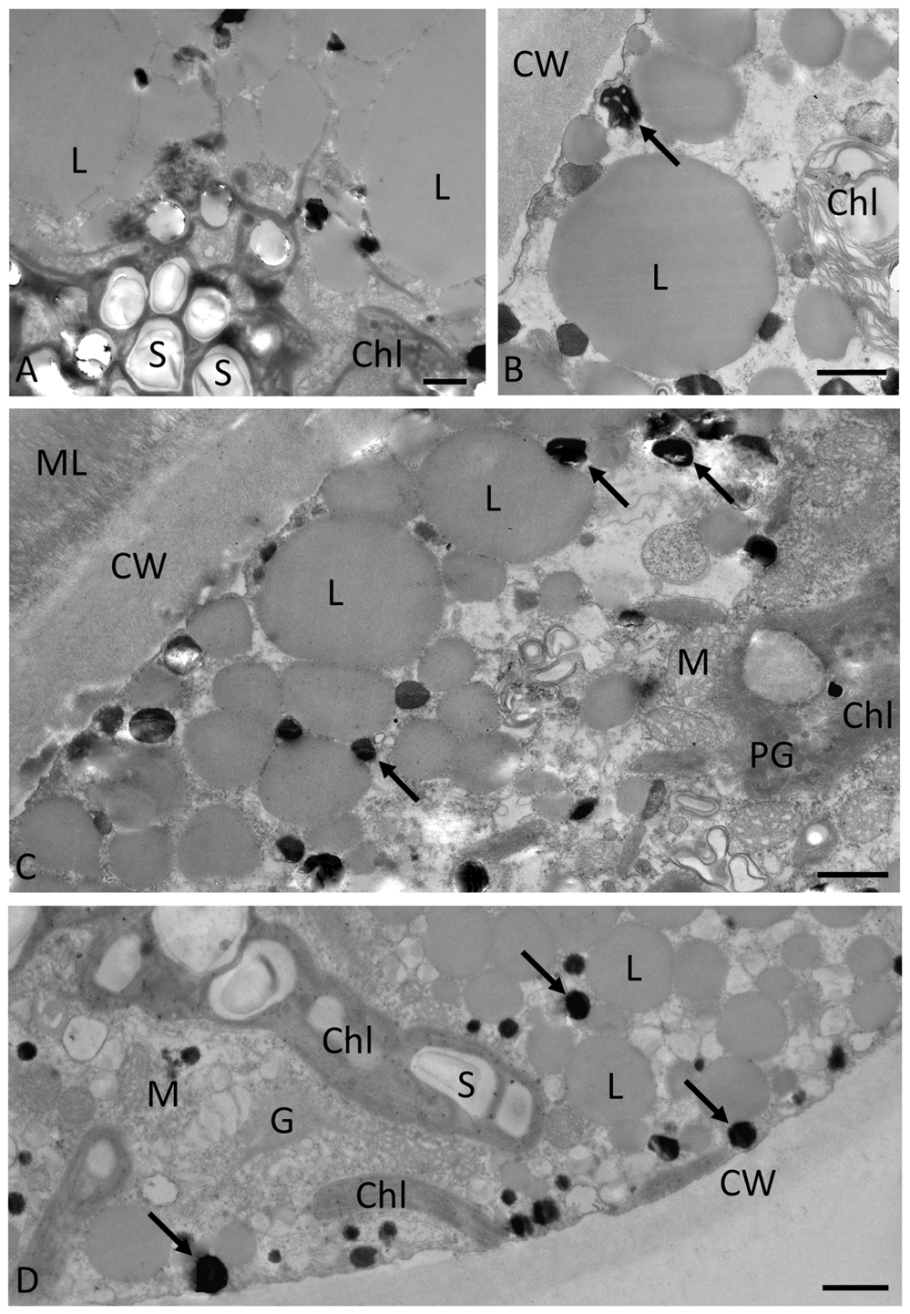
Transmission electron micrographs of pre-akinetes prior to desiccation. **A:** *Zygnema* sp. B. Large lipid bodies and chloroplast with starch grains are indicated. **B:** *Zygnema* sp. C. Lipid bodies and electron-dense particles (arrow) are indicated. **C:** *Zygnema* sp. E. Lipid bodies in the cell periphery, electron-dense particles (arrows) and cell walls with a fibrillose mucilage layer are marked. **D:** *Zygnemopsis* sp. L. Chloroplast lobes with starch grains, electron-dense particles (arrows), and lipid bodies are marked. All cultures were cultivated on agar medium without nitrate (A BBM-N) for 9 weeks. Abbreviations: Chl: chloroplast; L: lipid body; M: mitochondrion; ML: mucilage layer; PG: plastoglobules; S: starch. Scale bars: 1 µm.

**Figure 7:**
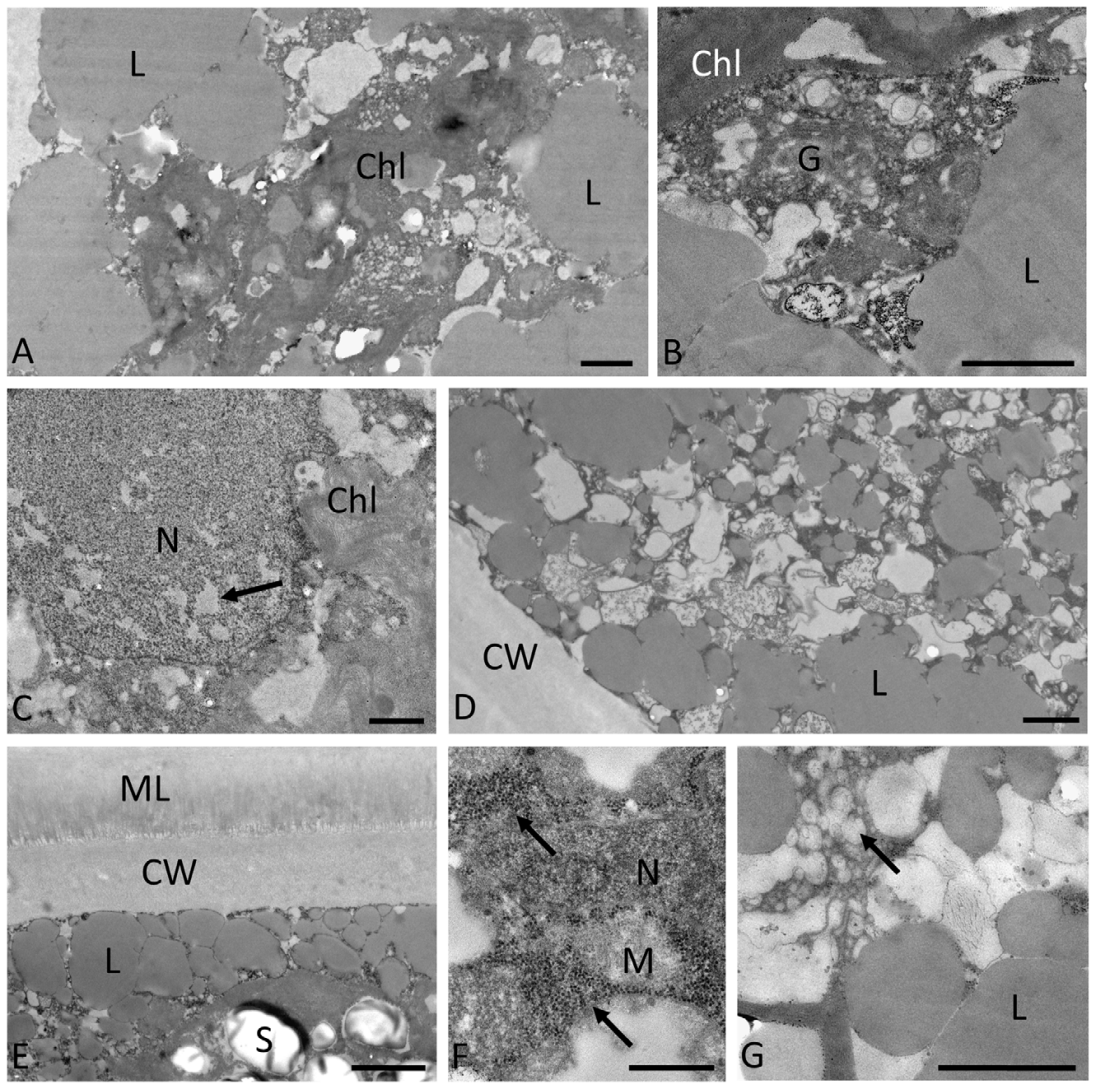
Transmission electron micrographs of pre-akinetes following desiccation for 2.5 hours at 86% rh. **A–C:** *Zygnema* sp. B; **D:** *Zygnema* sp. C; **E**–**F:** *Zygnema* sp. E; and **G:** *Zygnemopsis* sp. L. Note the following features: **A:** dense structure of the chloroplast and fusions of lipid bodies; **B:** dense structure of Golgi body and chloroplast; **C:** nucleus with less electron-dense areas of heterochromatin (arrow); **D:** accumulation of lipid bodies in the cell periphery; **E:** starch grains, lipid bodies in the cell periphery and the cell wall covered by a fibrillose mucilage layer; **F:** accumulations of ribosomes (arrows) next to the nucleus; and **G:** accumulation of vesicles and small electron translucent compartments next to the lipid bodies. Abbreviations: Chl: chloroplast; G: Golgi body; L: lipid body; M: mitochondrion; ML: mucilage layer; N: nucleus; S: starch. Scale bars: A–D and G: 1 µm; E: 2 µm; F: 0.5 µm.

TEM was performed on cultures cultivated for 9 weeks on A BBM-N (Fig. 6) and on the same cultures following desiccation for 2.5 hours at 86% rh (Fig. 7). All four strains investigated (*Zygnema* sp. B, Fig. 6A; *Zygnema* sp. C, Fig. 6B; *Zygnema* sp. E, Fig. 6C and *Zygnemopsis* sp. L, Fig. 6D) had similar ultrastructural appearances. In all cases, large lipid bodies with a diameter of several µm were apparent in the cell periphery (Fig. 6A–D). Accumulation of starch grains indicated the chloroplasts were still active (Fig. 6A and D). Electron-dense granules between the lipid droplets were observed in all the investigated strains. The chloroplasts occasionally contained plastoglobules (Fig. 6C). The cell walls were homogenous, about 2–4 µm thick and, particularly in *Zygnema* sp. E, were covered with a fibrillose mucilage layer.

Following desiccation for 2.5 hours, the cytoplasm appeared denser (Fig. 7A and B) but individual organelles, such as the chloroplasts (Fig. 7A) and Golgi bodies (Fig. 7B), were still visible. The peripheral lipid bodies tended to accumulate and form large structures by fusion (Fig. 7A, B and D). The nucleus was still intact; however, occasionally less electron-dense areas were observed in the heterochromatin (Fig. 7C). The marked mucilage layer outside the cell wall in *Zygnema* sp. E was still visible (Fig. 7F). The chloroplasts contained starch grains (Fig. 7F), as well as plastoglobules. Particularly dense accumulations of ribosomes surrounding the nucleus were observed (Fig. 7F). In *Zygnemopsis* sp. L, the cytoplasm contained numerous smaller vesicles as well as compartments with a medium electron-dense contrast (Fig. 7G).

## Discussion

The aims of this study were to investigate the culture conditions that lead to the formation of pre-akinetes in *Zygnema* spp. and *Zygnemopsis* sp., and to study their tolerance of desiccation under controlled laboratory conditions. We confirmed the hypothesis that nitrogen starvation induces pre-akinete formation, as a greater proportion of pre-akinetes developed in both agar-solidified and liquid BBM-N cultures than in regular BBM cultures. Moreover, a ‘hardening process’ or acclimation, resulting in desiccation tolerance, can be induced in pre-akinetes of filamentous Zygnematophyceae under specific cultivation conditions. This is the first report on induction of desiccation tolerance by cultivation under conditions of nitrogen-limitation and mild desiccation stress in polar hydro-terrestrial microalgae.

### Phylogenetic position

Both the Antarctic strains (C and E) shared the same *rbc*L sequence, which indicates that they are likely to be different isolates of the same species of *Zygnema*. This was an unexpected result, as they originated from different Antarctic islands: strain C was isolated from a seepage on James Ross Island whereas strain E was isolated from a meltwater pool on King George Island. It can be assumed that both sites, obviously shallow and temporary, provide similar ecological conditions. These two strains were also very similar in morphology and performance in desiccation experiments. Phylogenetic analyses placed them among species characterized by brown mesospore layer of specialized cells [41]. The occurence of brown akinetes in cultures of *Zygnema* sp. C is very well in accordance with the phylogenetic position and supports the importance of this trait for infrageneric classification of *Zygnema*.

The Arctic strain *Zygnema* sp. B, although very closely related to strains C and E, possibly belongs to a different species, as assessed by differences in the *rbc*L sequence (seven base-pair changes relative to the Antarctic strains), geographical origin, and characteristic phenotypic features, including filament width and stress tolerance under experimental conditions. The phylogenetic relationships are not reflected by geographical origin because both Arctic and Antarctic strains were found to be closely related to a *Z. irregulare* strain (RS012) which was isolated from California [41]. Moreover, another *Zygnema* sp. (strain G) isolated from Svalbard belonged to a different major clade within the genus, separate from strains B and E [37].

The identity of the *Zygnemopsis* sp. L strain was not known before the application of the molecular analysis. In its vegetative state, *Zygnemopsis* can be easily confused with *Zygnema* because of their similar chloroplast morphology. This study is the first report, to our knowledge, of the occurrence of *Zygnemopsis* sp. in Arctic samples. Interestingly, the two genera are not phylogenetically closely related [40].

### Starvation and pre-akinete formation

It has long been known that some microalgae survive unfavorable conditions by entering stationary phase and forming so-called akinetes [28]–[30]. Such senescent cells are usually characterized by thickened cell walls and a lower pigment content, and are filled with storage material [21], [28], [30]. In our cultures, they appeared darker and brownish in color, in comparison to vegetative cells from fresh cultures where the cells showed a high degree of vacuolization and a clearly stellate chloroplast [31], [37]. Even after 9 weeks of cultivation, our BBM cultures still contained a mixture of all types of cells from normal vegetative cells through to stationary phase cells (pre-akinetes). Nevertheless, when strong nitrogen starvation was applied by 9 weeks growth in nitrogen-free medium (both A BBM-N and L BBM-N), all cells appeared as pre-akinetes. Pre-akinetes with distinctly thickened cell walls were most readily produced in BBM-N cultures of the Antarctic strains *Zygnema* sp. C and *Zygnema* sp. E.

TEM demonstrated the ultrastructural changes that accompanied nitrogen starvation and the formation of pre-akinetes. Our observation is in accordance with an earlier study [28], which showed a massive accumulation of lipids in the cell cortex and a reduction of chloroplast lobes. In contrast to pre-akinetes, TEM revealed larger vacuoles in vegetative cells from young cultures of *Zygnema* sp. B and *Zygnema* sp. E [31], [37]. This is in good concordance with observations on two phylogenetically distant *Zygnema* spp. isolated from alpine regions, where younger cells showed large vacuolization, whereas older cultures (up to 15 months) showed cells filled with lipids and other storage compounds [33].

The accumulation of lipids typically accompanies nitrogen limitation in algae [30], [35], [36]. Lipid composition also changes with starvation; this has been described at the transcriptomic level in *Chlorella vulgaris* UTEX 395 [50]. Moreover, air drying has also been found to be a potential trigger for stimulation of triacylglycerol biosynthesis in *Chlorella kessleri* [51]. Recently, a transcriptomic study of *Klebsormidium* (Klebsormidiophyceae, Streptophyta) demonstrated that gene expression involved in energy production (e.g. citrate cycle, glycolysis, fatty acid degradation) was mainly up-regulated under desiccation stress [52]. The biochemical composition of zygnematophycean pre-akinetes remains to be investigated in detail and such findings will provide deeper insights into the acclimation process. The observed accumulation of lipids and other storage compounds are expected to be beneficial for tolerating desiccation stress.

We first studied directly the ultrastructure of pre-akinetes in the desiccated state. Surprisingly, their cell walls appeared rather unimpaired, indicating a different strategy to that used by *Klebsormidium* spp., which possess very flexible cell walls that allow massive shrinkage during desiccation [25], [53]. In general, a condensation of the cytoplasm was observed in all strains following desiccation, as previously reported in other streptophyte green algae [25], [53]. The slight changes in heterochromatin appearance and the accumulation of ribosomes, particularly around the nucleus, can be regarded as common stress reactions. Moreover, the mucilage layer outside of the cell walls, observed particularly in *Zygnema* sp. E, may additionally contribute to desiccation tolerance. This layer is likely composed of pectic components as described in *Z. irregulare* [32] and can contribute by its charged carboxyl groups significantly to better water holding capacities. A similar mucilage layer has been observed in a natural *Zygnema* population from an Arctic habitat [18], [21].

Besides pre-akinetes, microalgae also produce other types of resistant cells that enable them to survive stresses. Several cell types have been described in *Zygnema* [10], but none have ever been reported from polar regions. In *Zygnema* sp. C cultures, mature *Zygnema* akinetes with brown mesospores rarely occurred, but their role in desiccation tolerance could not be proven in this study. Nevertheless, desiccation tolerance of a very closely related strain of *Zygnema irregulare* isolated from California was recently investigated [32]. Similarly to our results, the author also reported the formation of both pre-akinetes (stationary phase-like cells) and akinetes (specialized cells with brown and lamellate cell walls) in one strain during prolonged cultivation on agar.

### Desiccation tolerance and drying rate

In our study, vegetative or morphologically intermediate cells, even if pre-grown on agar, did not survive any of the desiccation treatments, indicating desiccation tolerance can be induced only in pre-akinetes. Thus, the recovery experiments reflect only the response of the pre-akinetes in both BBM and BBM-N cultures. Nevertheless, Figure 5 clearly demonstrates that pre-akinetes from starved cultures (BBM-N) of the three *Zygnema* strains were, in general, more resilient than those from BBM, as they survived more rapid desiccation. By contrast, in *Zygnemopsis* sp. L, nitrogen depletion resulted in severe damage and produced very low initial values of the effective quantum yield.

The pre-akinetes, which developed during the period of experimental pre-cultivation, tolerated, in most cases, conditions of moderate desiccation (86% rh and ‘very slow’ desiccation). Nevertheless, the pre-akinetes were in some cases able to survive rapid desiccation at a relative humidity of 10% after previous acclimation by slow desiccation (induced either by controlled desiccation at high relative humidity or by pre-cultivation on agar). These data agree very well to field observations where slow desiccation enhanced osmotic stress tolerance [21]. Our results also suggest that the desiccation tolerances of *Zygnema* spp. and *Zygnemopsis* sp. are not constitutive, but detailed proteomic or metabolomic analyses will be needed to test this hypothesis.

Moreover, recovery of photosynthetic activity and viability during rehydration were clearly dependent on speed of drying, i.e., the slower the desiccation, the better the level of survival. The desiccation rate is in general an important factor affecting the survival and recovery after rehydration. For example, *Klebsormidium dissectum* showed much faster recovery of the optimum quantum yield when desiccated at 100% rh than at 55% or 5% rh [25]. The same effect was observed in a common lichen photobiont, *Trebouxia ericii* (now *Asterochloris ericii*; [27]); even the ultrastructural injuries were more pronounced after fast desiccation [54]. Similarly, fast drying (at 50% rh) was lethal for the moss *Fontinalis antipyretica*, which survived when desiccated at 95% rh [55].

Filamentous Zygnematophyceae typically form extensive mats and produce mucilage that provides a natural protection against quick dehydration and allows sufficient time for hardening [2], [18], [19]. Thus, it can be assumed that the rapid desiccation applied in our experiments does not appear under natural conditions. Nevertheless, the ability to survive strong desiccation stress is still important for these algae; the akinetes liberated by filament fragmentation are no longer protected within mats but instead serve as ideal airborne propagules for dispersion by means of asexual reproduction [13], [28].

We showed that in addition to the drying rate [27], duration of desiccation [25] and light conditions during desiccation [26], the age and state of the culture also play an important role in survival and recovery after desiccation. This finding has an important consequence for laboratory experiments that investigate desiccation tolerance. Numerous experimental desiccation studies on various algal cultures have been published so far (for a recent review see [2]). Typically, log-phase cultures are used in such experiments, but we suggest that older or starved cultures should also be tested. Morphology of microalgae under natural conditions often resembles such old cells [30], [56], [57] and, therefore, it is different from that under optimal culture conditions. Thus, hardened stationary phase cells may reflect natural conditions better than log-phase cultures and the comparison of their responses will contribute to an enhanced understanding of an alga's real capacity for desiccation tolerance.

### Light stress during desiccation

The survival capacity in a desiccated state is strongly influenced by other stress factors, the most important of which is light. Homoiochlorophyllous plants (most algae) retain chlorophyll when desiccated, which gives them the ability to recover their physiological activity quickly after rehydration [58]. There is, however, a strong danger of photodamage and photobleaching during desiccation because the chlorophyll molecules can still be excited when illuminated, but the energy produced cannot be transferred through photochemistry, leading to the production of reactive oxygen species [23]. Thus, algae desiccated under illuminated conditions are much less viable after rehydration than those desiccated in darkness [26].

*Zygnema* and *Zygnemopsis* cells retained their chloroplasts following desiccation, although they were partly altered in pre-akinetes. The characteristic lobes of the stellate chloroplasts were reduced and photosynthetic activity diminished. A similar reduction in chloroplasts has been observed in *Klebsormidium rivulare* akinetes [30]. We hypothesize that the reduction of chloroplast size is a general strategy that could reduce light stress during desiccation.

In our experiments, the cultures were continuously illuminated at a light intensity of 50 µmol m^−2^s^−1^. Such illumination is not stressful, as it is far below the prevailing irradiance in Svalbard during the summer, and well below the photoinhibition limits determined for Arctic and Antarctic *Zygnema* strains, which are around 500 µmol m^−2^s^−1^ [31]. Nevertheless, it is possible that the survival capacity of the strains tested would be even better when desiccated in darkness, but such a situation does not occur under natural conditions. However, in nature, self-shading, which reduces the light availability for cells from lower layers drastically, might be an important factor in mat-growing organisms like *Zygnema*. A similar strategy has been reported in another polar hydro-terrestrial alga *Prasiola* sp. [59].

Keeping our samples illuminated during desiccation and rehydration was necessary to allow regular measurements of the effective quantum yield, even at 2 minute intervals. This fluorescence parameter is measured under light conditions, to which the photosynthetic apparatus is adapted, and gives a good estimation of photosynthetic activity [60].

### Desiccation tolerance in different Zygnematophyceae

All the strains used in this study survived desiccation under certain circumstances, but differences in stress resistance were found. The Antarctic *Zygnema* sp. C and *Zygnema* sp. E were best adapted to stressful conditions, as they were the only strains to survive direct exposure to 10% rh following pre-cultivation on A BBM-N. The Arctic strain *Zygnema* sp. B was less stress-resistant than the Antarctic strains; it survived moderate desiccation at 86% rh. Interestingly, an analysis of the osmotic stress tolerance of two of the *Zygnema* strains used in this study (B and E) found that *Zygnema* sp. B had a lower (more negative) osmotic potential (approximately 600 mM sorbitol, ψ −1.67 MPa) than *Zygnema* sp. E (approximately 300 mM sorbitol, ψ −0.8 MPa) [31]. This result suggests that *Zygnema* sp. B may be more resistant to osmotic stress; however, the study was performed with young cultures [31].

Very slow desiccation led to acclimation in all *Zygnema* strains, as reflected by their better rate of survival following a subsequent transfer to 10% rh. By contrast, *Zygnemopsis* sp. L did not survive in 10% rh even after such an acclimation period, which indicates that it is less desiccation-tolerant than the *Zygnema* strains used in this study. Generalizing from this to the whole genera must be strictly avoided, because desiccation stress tolerance was not thoroughly investigated in a sufficient number of strains.

Nevertheless, it appears that hardened pre-akinetes (starved vegetative cells, rather than specialized stages) play a key role in stress tolerance in conditions where production of specialized dormant stages is largely suppressed. However, pre-akinete formation and desiccation tolerance are not exclusively characteristic for strains isolated from polar habitats. A similar strategy was observed in *Zygnema* sp. isolated from a humid subtropical climate (Austin, Texas) [28], in *Z. stellinum* from a temperate continental climate in Belarus [34] and two distantly related *Zygnema* spp. (SAG 2418 and 2419) isolated from alpine habitats [33].

Stress tolerance is a widespread phenomenon in Zygnematophyceae and many species occur in extreme environments, such as the surface of glaciers [14], [15]. Other members of the group, such as various desmids [12], [61] or *Zygogonium ericetorum* [62]–[65], occur in ephemeral habitats that dry out regularly. Desiccation tolerance in Zygnematophyceae is of particular interest because of the close relationship between this class and all land plants [8], [9]. The mechanisms that govern desiccation tolerance in this potential sister group [6] and thus allow successful colonization of the terrestrial environment are expected to be shared by other plants.

## Supporting Information

Figure S1 **Light micrographs of the experimental strains pre-cultivated in liquid medium for 9 weeks.** **A**–**D:** cultures grown in regular L BBM medium; and **E**–**H:** cultures grown in L BBM-N medium. The pictures were taken prior to the desiccation experiments. Scale bars: 10 µm.
(TIF)

Table S1 **Viability of cultures pre-cultivated in liquid medium following 48 hours of rehydration in water (L BBM and L BBM-N cultures).** −: No living cells observed; +: <5% living cells; ++: 5–50% living cells; +++: 50–100% living cells.
(DOCX)
