# Supplementary figures and images for: Nitrogen Limitation and Slow Drying Induce Desiccation Tolerance in Conjugating Green Algae (Zygnematophyceae, Streptophyta) from Polar Habitats
Source: PLoS One. 2014 Nov 14;9(11):e113137. doi: 10.1371/journal.pone.0113137 (PMC4232603; doi:10.1371/journal.pone.0113137)

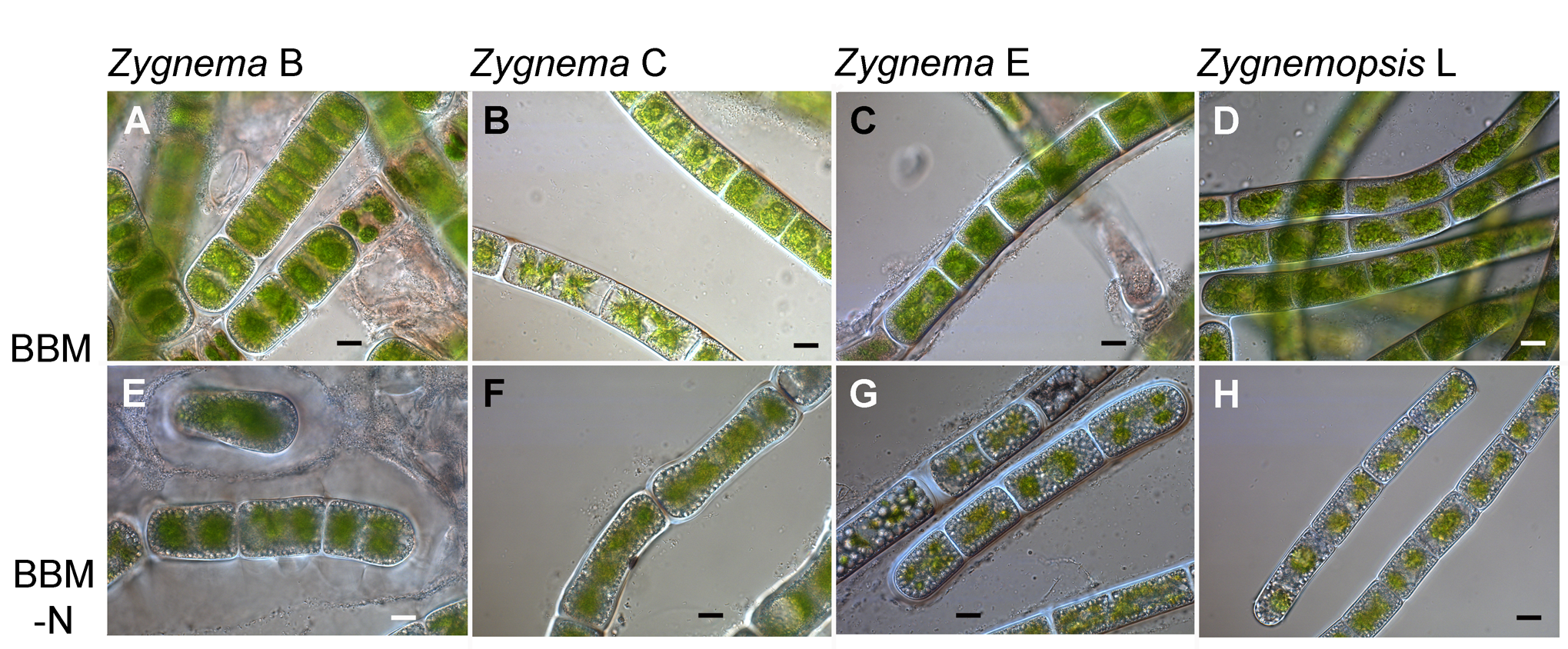

Supplement: Figure S1 — Light micrographs of the experimental strains pre-cultivated in liquid medium for 9 weeks. A–D: cultures grown in regular L BBM medium; and E–H: cultures grown in L BBM-N medium. The pictures were taken prior to the desiccation experiments. Scale bars: 10 µm. (TIF) [file pone.0113137.s001.tif]
