# Supplementary material for: Nitrogen Limitation and Slow Drying Induce Desiccation Tolerance in Conjugating Green Algae (Zygnematophyceae, Streptophyta) from Polar Habitats
Source: PLoS One. 2014 Nov 14;9(11):e113137. doi: 10.1371/journal.pone.0113137 (PMC4232603; doi:10.1371/journal.pone.0113137)
Supplement: Table S1 — Viability of cultures pre-cultivated in liquid medium following 48 hours of rehydration in water (L BBM and L BBM-N cultures). −: No living cells observed; +: <5% living cells; ++: 5–50% living cells; +++: 50–100% living cells. (DOCX) [file pone.0113137.s002.docx]

| **Strain** | **B** | | **C** | | **E** | | **L** | |
| --- | --- | --- | --- | --- | --- | --- | --- | --- |
| **Culture type** | **BBM** | **BBM-N** | **BBM** | **BBM-N** | **BBM** | **BBM-N** | **BBM** | **BBM-N** |
| 10% rh | - | - | - | - | - | - | - | - |
| 86% rh | - | + | + | + | + | ++ | + | - |
| 86% rh + BBM | ++ | +++ | ++ | ++ | + | ++ | +++ | ++ |
